# Outcomes of Mechanical Thrombectomy in Patients With Neurological Disorders: A National Inpatient Sample Database Analysis

**DOI:** 10.7759/cureus.54063

**Published:** 2024-02-12

**Authors:** Mohammad El-Ghanem, Hael Abdulrazeq, Leonardo Brasiliense, Hamza Abbad, Pedro Aguilar-Salinas, Fawaz Al-Mufti, Travis Dumont

**Affiliations:** 1 Neurology, Hospital Corporation of America (HCA) Houston Healthcare Northwest, Houston, USA; 2 Neurosurgery, Brown University, Providence, USA; 3 Neurological Surgery, University of Arizona College of Medicine - Tucson, Tucson, USA; 4 Neurosurgery, University of Arizona, Tucson, USA; 5 Neurosurgery, Westchester Medical Center, Westchester, USA

**Keywords:** endovascular surgery, mechanical thrombectomy, stroke

## Abstract

Introduction: Mechanical thrombectomy (MT) has changed the standard of care for patients presenting with acute ischemic stroke (AIS). The window of treatment has significantly increased the number of patients who would benefit from intervention and operators may be confronted with patients harboring preexistent neurological disorders. Still, the epidemiology of patients with AIS and neurological disorders has not been established.

Methods: This is a retrospective study, which utilizes data from the National Inpatient Sample (NIS) between 2012 and 2016. Patients with the major neurological comorbidities in the study were included: Alzheimer’s dementia (AD), Parkinson’s disease (PD), amyotrophic lateral sclerosis (ALS), multiple sclerosis (MS), and myasthenia gravis (MG). These patients were divided into groups and analyzed based on discharged home status, length of hospital stay (LOS), and inpatient mortality. These outcomes were also compared between patients who underwent MT versus those who did not.

Results: In this study, 460,070 patients with AIS were identified and included. MT was performed less often when the patient had a neurological diagnosis compared to those without a neurological disease (p<0.0001). However, patients with AIS who have underlying neurological disorders such as AD, PD, and MS have shown similar outcomes after MT to those who do not have these disorders.

Conclusion: Patients with preexisting neurological disorders were less likely to undergo MT. Further studies are required to elucidate the implications of having a neurological disorder in the setting of an AIS.

## Introduction

Mechanical thrombectomy (MT) has become the most innovative standard of care for patients with acute ischemic stroke (AIS) caused by large vessel occlusion (LVO) in the anterior cerebral circulation [1,2]. Studies have shown greater outcomes in patients treated promptly with MT in comparison to patients provided with medical treatment alone, regardless of the patient characteristics such as age, sex, baseline stroke severity, site of occlusion, or use of alteplase [3,4]. With more than 800,000 strokes occurring annually, the potential for performing MT in the proper timing can have a substantial effect, as time to treatment in patients with AIS is of the essence in achieving the best outcome possible. Shorter times to MT have been associated with a higher modified Rankin Scale (mRS) at three months, lower mortality rates, and a greater percentage of patients being discharged home [5-9].

With regard to age specifically, the Highly Effective Reperfusion evaluated in the Multiple Endovascular Stroke Trials (HERMES) study, which was a meta-analysis of randomized controlled trials of MT, showed that despite the benefits of MT in patients older than 80, age was found to be an independent factor that was associated with worse outcomes following MT, possibly due to age acting as a surrogate for unfavorable anatomy for MT [3]. Imahori et al. further investigated this by looking at patients aged 80 years or older and compared their functional outcomes with those younger than 80, and found no significant correlation between age and outcome of MT [9]. Of note, the patients in this study included those who had lived independently and had an mRS ≤2.

Historically, variable correlations between AIS and coexisting comorbidities undergoing MT have been discussed; however, there is limited data discussing patients who have suffered AIS with underlying neurological disorders, especially in the older age population. This is important as there may exist treatment bias for patients with comorbidities in this age group, and MT may have been deemed futile in these patients due to presumed poor functional outcomes. To our knowledge, there have not been studies that compare the outcomes of MT in patients with disorders such as Alzheimer’s dementia (AD), Parkinson’s disease (PD), amyotrophic lateral sclerosis (ALS), multiple sclerosis (MS), or myasthenia gravis (MG). In this study, we aim to examine those neurologic comorbidities and their relation to outcomes of MT in stroke patients.

## Materials and methods

Study design and data collection

A retrospective study was conducted utilizing the National Inpatient Sample (NIS) database analyzing data between 2012 and 2016. Our study analyzed data from the NIS, the largest all-payer inpatient care database created by the Agency for Healthcare Research and Quality. This is a compendium of yearly discharge data from more than 1,000 short-term and nonfederal hospitals, which represents approximately a stratified sample of 20% of hospitals in the US. Each hospitalization is treated as an individual entry in the database and is coded with one principal diagnosis, up to 14 secondary diagnoses, and 15 procedural diagnoses associated with the hospital stay. Information available from the NIS also includes patient volume and whether the admitting hospital is a teaching or a non-teaching facility. Discharge weights are provided to facilitate the projection of national estimates, along with information necessary to calculate the variance of estimates. Further details about discharge weights can be accessed on the website (https://www.hcup-us.ahrq.gov/nisoverview.jsp).

All patients with the following major neurological comorbidities were included: AD, PD, ALS, MS, and MG; they were identified by referring to the International Classification of Disease, 9th Revision, Clinical Modification (ICD-9-CM). Primary or secondary diagnosis codes to identify patients were screened using the following codes: AD 331.0, PD 332.0, ALS 335.20, MS 340, and MG 358. Primary or secondary procedure codes were screened for cerebral MT: AD G30, PD G20, ALS G12.21, MS G35, MG G70, and MT ICD-10-PCS 03CG3ZZ. ICD-9 study variables extracted from the database include patient mortality and length of stay.

Endpoints

The primary endpoints of this study were to assess the rates of discharge home, length of hospital stay (LOS), and inpatient mortality between patients with major neurological comorbidities who underwent MT and those who did not. Comparison between groups was performed based on each neurological disorder: AD, PD, MS, ALS, and MG.

Statistical analysis

Descriptive data were presented as absolute values and percentages for categorical variables. Patients were categorized into two groups: patients who underwent MT versus patients without MT. The chi-square test or Fisher’s exact test was used for comparisons between categorical variables as appropriate. All reported p-values were two-tailed and were considered statistically significant when p <0.05. Statistical analysis was performed by using SAS version 9.4 (SAS Institute Inc., Cary, NC).

## Results

Study population

In total, 460,070 patients with AIS were identified. Of these patients, there were 10,775 with AD (2.3%), 5,630 with PD (1.2%), 1,430 patients with MS (0.31%), 56 with ALS (0.012%), and nine with MG (0.001%). Overall, 7,529 (1.63%) patients underwent MT (Table 1).

**Table 1:** Thrombectomy outcomes in patients with neurological diseases (AD, MS, PD, ALS). LOS: length of hospital stay; MT: mechanical thrombectomy; AD: Alzheimer’s dementia; MS: multiple sclerosis; PD: Parkinson’s disease; ALS: amyotrophic lateral sclerosis.

	No MT		MT	
	No neurological disease	Neurological disease	No neurological disease	Neurological disease
N	434915	17626	7411 (1.7%)	118 (0.7%)
Discharged home	51.2%	31.9%	26.2%	15.3%
LOS	4.84%	4.84%	8.79%	8.76%
Inpatient mortality	4.0%	4.6%	14.4%	14.4%

Data analysis

Tables 2-6 summarize the findings for patients with neurological disorders presenting with AIS and compare their outcomes according to MT status.

**Table 2:** Thrombectomy outcomes in patients with Alzheimer’s disease AD: Alzheimer’s disease; LOS: length of hospital stay; MT: mechanical thrombectomy.

	No MT			MT		
	No AD	AD	p-Value	No AD	AD	p-Value
N	441822	10719		7473	56	
Discharged home	51%	29%	<0.0001	26%	9%	0.0034
LOS	4.84%	4.92%	0.17	8.78%	6.5%	0.077
Inpatient mortality	4%	5%	<0.0001	14%	11%	0.43

**Table 3:** Thrombectomy outcomes in patients with Parkinson’s disease LOS: length of stay; MT: mechanical thrombectomy; PD: Parkinson’s disease.

	No MT			MT		
	No PD	PD	p-Value	No PD	PD	p-Value
N	446963	5578		7477	52	
Discharged home	51%	32%	<0.0001	26%	21%	0.42
LOS	4.84%	4.91%	0.39	8.77%	7.31%	0.27
Inpatient mortality	4%	5%	0.044	14%	19%	0.31

**Table 4:** Thrombectomy outcomes in patients with multiple sclerosis LOS: length of stay; MS: multiple sclerosis; MT: mechanical thrombectomy.

	No MT			MT		
	No MS	MS	p-Value	No MS	MS	p-Value
N	451125	1416		7515	14	
Discharged home	50%	50%	0.64	26%	21%	0.69
LOS	4.84%	4.774%	0.68	8.77%	5.429%	0.19
Inpatient mortality	4%	2%	0.0007	14%	7%	0.44

**Table 5:** Thrombectomy outcomes in patients with amyotrophic lateral sclerosis ALS: amyotrophic lateral sclerosis; LOS: length of hospital stay; MT: mechanical thrombectomy.

	No MT			MT		
	No ALS	ALS	p-Value	No ALS	ALS	p-Value
N	452485	56		7529	0	
Discharged home	50%	50%	0.94	26%	N/A	N/A
LOS	4.84	4.32	0.52	8.76	N/A	N/A
Inpatient mortality	4%	7%	0.23	14%	N/A	N/A

**Table 6:** Thrombectomy outcomes in patients with myasthenia gravis LOS: length of hospital stay; MG: myasthenia gravis; MT: mechanical thrombectomy.

	No MT			MT		
	No MG	MG	p-Value	No MG	MG	p-Value
N	452532	9		7529	0	
Discharged home	50%	n/a	n/a	26%	n/a	n/a
LOS	4.84	n/a	n/a	8.76	n/a	n/a
Inpatient mortality	4%	n/a	n/a	14%	n/a	n/a

Overall, MT was performed less often in patients with preexisting neurological disorders (0.65%; 118/17,900) compared to other patients (1.67%; 7,411/442,170). Refer to Table 1 for further details.

Patients with AD who did not undergo MT had a significantly lower rate of being discharged home compared to patients without neurological disorders (29% vs. 51%, p<0.0001), as well as a higher inpatient mortality rate (5% vs. 4%, p<0.001). There was no significant difference in LOS between these two groups. Patients with AD who underwent MT were less likely to be discharged home compared to those without AD (9% vs. 26%, p<0.0034). LOS and inpatient mortality rates were lower in this group, yet this was not statistically significant (Table 2).

Patients with PD were less likely to be discharged home, especially if the patient did not undergo MT (32% vs. 51%, p<0.0001). In terms of LOS, no statistical difference was found. Regarding inpatient mortality, patients with PD had an overall higher rate regardless if patients underwent MT (Table 3).

In patients with MS, only 14 of those underwent MT and no statistical difference was found in the clinical endpoints with similar rates in both groups (Table 4).

Regarding patients with MG or ALS, no patients underwent MT. These two groups were also smaller (56 ALS patients and nine MG patients). No comparisons could be made in the MG group. For ALS patients with AIS, there was a higher inpatient mortality rate, shorter LOS, and similar rates of being discharged home compared to those without ALS, and these differences were found to be not significant (Table 5).

## Discussion

Endovascular intervention for patients with AIS with preexisting neurological disorders was found to be lower compared to other patients (0.65% vs. 1.67%). Among all patients who underwent MT, inpatient mortality rates did not differ between patients who had these disorders and those who did not (14.4% for each). These findings suggest that the presence of these neurological disorders in patients with AIS should not preclude them necessarily from being offered MT. Specifically, patients with AD and PD were found to have longer in-hospital LOS when they did not undergo MT. This could potentially be attributed to persistent neurological deficits in those patients who do not undergo MT. These patients may require a higher and more intense level of physical therapy and rehabilitation, and the need for discharge to an acute rehab or skilled nursing facility. Additionally, when MT was not performed, there was a higher inpatient mortality rate. This information could lead to further studies in the future that study outcomes in these groups of patients. This may be of value in counseling families about their expectations in patients with neurological disorders who are being offered MT. This may also address any underlying bias from providers toward these patients with regard to the perceived futility of intervention in these patient populations. However, the proportion of patients in this analysis who had MG or ALS was much smaller than the other diseases mentioned in this study, and as such, no significant differences could be identified in outcomes. Only 14 patients with MS underwent MT in this study, and so the sample size was small; however, no significant differences were found in the studied endpoints. This seems to suggest that MT when performed in patients with MS has similar outcomes to those patients without MS, which suggests that the presence of MS should not be a contraindication to receiving MT.

Recognizing an underlying bias toward the treatment of these groups of patients is important, as there have been studies that suggest that physicians are prone to allowing their implicit biases affect their clinical judgment [10]. LVO is a time-sensitive emergency, and providers have to make decisions regarding treatment under stressful conditions. As such, recognizing that the presence of these neurological disorders is not a contraindication to thrombectomy is critical, and providers need to be conscious of this to avoid any decisions that may deny a good candidate for thrombectomy from their treatment.

To our knowledge, this is the first study to assess the epidemiology of patients with preexisting neurological disorders who underwent MT. Other studies have reported outcomes in patients with AIS as well as cancer or diabetes [11,12].

Limitations

Our study has several limitations due to its retrospective nature. The NIS dataset is limited in patient information. No casualty or correlation may be inferred based on the data analyzed for patient selection in the MT group. However, NIS is of benefit in providing a large set of patients nationwide. Another limitation is the time period from which these patients were selected (2012-2016), preceding the pivotal trials, which favored thrombectomy for patients with LVO [3].

## Conclusions

Patients with preexisting neurological disorders were less likely to undergo MT. Further studies are required to elucidate the implications of having a neurological disorder in the setting of an AIS.
